# Impact of foot progression angle on spatiotemporal and plantar loading pattern in intoeing children during gait

**DOI:** 10.1038/s41598-024-65422-5

**Published:** 2024-06-22

**Authors:** Yuqing Cao, Hanjie Zhuang, Xinhui Zhang, Ruoyi Guo, Haotian Pang, Pengfei Zheng, Hang Xu

**Affiliations:** 1 School of Medical Imaging, Xuzhou Medical University, Xuzhou, China; 2 Department of Orthopaedic Surgery, Children’s Hospital of Nanjing Medical University, Nanjing, China

**Keywords:** Foot progression angle, Plantar pressure, Intoeing, Biomechanics, Gait analysis, Disease prevention, Paediatrics, Paediatric research

## Abstract

Intoeing in children is a common parental concern, but our understanding of the impact of foot progression angle (FPA) in these children leaves remains limited. This study examines the relationship between FPA and plantar loading pattern, as well as gait symmetry in children with intoeing. The sample included 30 children with intoeing caused by internal tibial torsion, uniformly divided into three groups: unilateral intoeing, bilateral mild intoeing, and bilateral mild-moderate intoeing. The relationship between FPA and plantar loading pattern, and gait symmetry within and among groups were assessed using dynamic pedobarographic and spatiotemporal data. Results indicated a significant correlation between FPA and peak pressure, maximum force, and plantar impulse in the medial and central forefoot, and also the medial and lateral heel zones for both bilateral intoeing groups. Significant differences were observed only in subdivided stance phase, including loading response, single support, and pre-swing phases, between the unilateral intoeing and bilateral mild intoeing groups. These findings suggest that FPA significantly affects the forefoot and heel zones, potentially increasing the load on the support structures and leading to transverse arch deformation. While children with intoeing demonstrate a dynamic self-adjustment capability to maintain gait symmetry, this ability begins to falter as intoeing becomes more pronounced.

## Introduction

Intoeing gait is often cited as a frequent cause for appointments at pediatric orthopedic clinics. The degree of worry among parents about this condition is considerable, stemming from fears of resultant trips and falls, impeded participation in sports, and even future long-term disabilities such as arthritis^1^. Intoeing gait can present unilaterally or bilaterally in a clinical setting, generally assessed by the foot progression angle (FPA), which is the longitudinal foot axis relative to the forward progression line during ambulation^2^. A positive FPA denotes an out-toeing angle, conversely, a negative FPA signifies an intoeing angle^3^. Prominent reasons contributing to an intoeing gait consist of increased femoral anteversion, internal tibial torsion, metatarsus adductus, or a mix of these three conditions^4^. Early detection and treatment of intoeing gait is critical due to its potential significant impact on a child's mobility and functionality^1,5^. Potential treatment plans comprise observation, physical therapy, bracing or in severe cases, surgery—depending upon the fundamental cause and severity of the condition.

The evaluation of rotational profiles' causes and severity can be executed through a physical examination. Excessive femoral anteversion can be diagnosed by observing an amplified projection of the femoral neck on the femoral shaft, while metatarsus adductus is revealed by a C-shaped curve present on the foot's lateral border. A negative thigh-foot angle is a frequent observation in children suffering from internal tibial torsion, which is considered the most common cause of intoeing^6,7^. However, the treatment necessity and management methods for intoeing gait continue to be topics of contention. An estimated 80% of cases witness spontaneous resolution prior to skeletal maturity^8,9^. Conservative and surgical treatments are recommended only when the intoeing gait persists into skeletal maturity and causes dysfunction. Nevertheless, conventional interventions such as casts, orthopedic shoes, or insoles have demonstrated limited effectiveness or appear to be ineffective^9^. Surgical procedures, while proven effective, carry the potential for significant complications^6,10^.

Pedobarography, which provides dynamic plantar loading information and gait spatiotemporal parameters over multiple steps, is essential for assessing patients with foot issues.Various studies have substantiated the association between changes in plantar pressure and a range of foot symptoms and issues, including hallux valgus, flexible flatfoot, and cavovarus foot deformity^11–13^. However, research focusing on plantar pressure during intoeing gait is still scarce and the link between lower-limb rotation and foot loading patterns needs further examination. Craxford et al. reported no significant link between plantar pressure type and lower-limb rotation in normal children^14^, but Chang et al. discovered a significant correlation between FPA and the medial forefoot impulse in children with neuromuscular diseases^15^. Several other studies propose that an externally rotated FPA tends to increase medial foot loading, while an internally rotated FPA moves the foot loading toward the lateral side^16,17^. Furthermore, children with intoeing have been observed to exhibit a larger step width and greater ankle dorsiflexion, but a smaller stride length during gait compared to typical children^18^.

To the best of our knowledge, the ramifications of the severity of intoeing on the gait and foot loading patterns have yet to be thoroughly investigated. Consequently, this study intends to investigate into the relationship between FPA and foot loading patterns, and inspect the changes in spatiotemporal variables during gait among children with differing severities of intoeing. Our specific hypotheses are as follows: (1) There is a substantial correlation between FPA and plantar loading in distinct foot regions; and (2) the gait spatiotemporal variables exhibit disparities based on the severity of intoeing. Potential results from this study could improve our understanding of the features of intoeing gait patterns and potentiate the establishment of individually-tailored treatments and interventions.

## Methods

### Participants

A total of 72 intoeing children, aged between 3 and 9 years, from the Children’s Hospital of Nanjing Medical University underwent dynamic pedobarographic examination using the plantar pressure plate (FDM2, Zebris Medical GmbH, Germany). Since the central nervous system and gait pattern only mature after the age of four^19,20^, only children aged five and above were included in the current study. A retrospective analysis of pedobarographic data and clinical examinations was conducted. Finally, 30 children most likely affected by internal tibial torsion were included. Criteria for inclusion were as follows: (1) an age range between 5 to 9 years; (2) unilateral or bilateral intoeing with an FPA ranging between 0 and negative 18 degrees; (3) no neuromuscular diseases; (4) no other foot, knee and hip problems. Approval was granted by the Institutional Review Board from Children’s Hospital of Nanjing Medical University (Approval number 202112127–1) and written informed consent was procured from their guardians of the children. All methods in this study were conducted according to the relevant guidelines and regulation of the Declaration of Helsinki.

### Group division

According to established literature, mild intoeing is identified when the FPA falls between 0 and negative 10 degrees, and moderate intoeing is recognized when the FPA fluctuates between negative 10 and 18 degrees^21^. In our study, the selected 30 intoeing children were divided equally into three groups (Table 1). Group A encompassed children with unilateral intoeing, exhibiting normal FPA in one limb and mild intoeing in the other. Group B was comprised of children presenting bilateral mild intoeing, while group C included children with bilateral intoeing, manifesting mild intoeing in one limb and moderate intoeing in the other. There were no significant difference in age, height, weight, and BMI among the three groups, as verified by a one-way ANOVA test.

**Table 1:** The demographic characteristics of three intoeing groups.

	Group A		Group B		Group C	
	Normal	Mild	Mild-left	Mild-right	Mild	Moderate
Gender,M/F	6/4		6/4		6/4	
Age (years)	6.10 ± 1.14		5.90 ± 0.90		6.00 ± 0.80	
Height (m)	1.17 ± 0.11		1.15 ± 0.08		1.18 ± 0.11	
Weight (kg)	21.81 ± 4.71		20.66 ± 3.70		22.63 ± 5.21	
BMI (kg/m^2^)	15.72 ± 0.92		15.47 ± 1.25		15.85 ± 1.46	
FPA (degree)	2.91 ± 2.17	− 2.20 ± 1.60	− 5.38 ± 1.75	− 4.86 ± 2.14	− 5.05 ± 2.41	− 12.38 ± 1.50

### Protocol

All participating children were assessed uniformly using a specific protocol within the Biomechanics and Motion Analysis Laboratory at Xuzhou Medical University. The dynamic plantar pressure and spatiotemporal parameters were measured at a frequency of 100 Hz utilizing a plantar pressure plate (FDM2, Zebris Medical GmbH, Germany), which was 212 cm in length and 60.6 cm in width, equipped with 15,360 capacitive pressure sensors. To accomplish the assessment, each child was instructed to look straight forward and walk barefoot at a natural pace along a 6-m walkway that crossed over the plantar pressure plate. Upon reaching the terminus of the walkway, they were directed to complete a 180-degree turn and proceed to walk over the walkway again. This procedure was reiterated until the expiration of 30 s.

### Data process

The array of sensor within the plantar pressure plate recorded the dynamic position and pressure as subject walks across it by the Zebris FDM 1.18 software (Zebris Medical GmbH, Isny, Germany), and visual map of each footprint was generated. Then, the FPA was calculated automatically by compared the longitudinal axis of the foot to the line of forward progression during walking, which served as the foundational criterion for the division of groups.

Additionally, a gait analysis report was also generated, encompassing spatiotemporal parameters and plantar loading particulars. The assessed spatiotemporal variables comprised step length and step width, normalized to the leg length based on the previous literature^22^. The stance phase (subdivided in loading response, single support stance, and pre-swing), swing phase and double support phase were expressed as percentages of gait cycle. Velocity (m/s), cadence (steps/min), and step time (s) were also included.

**Figure 1:**
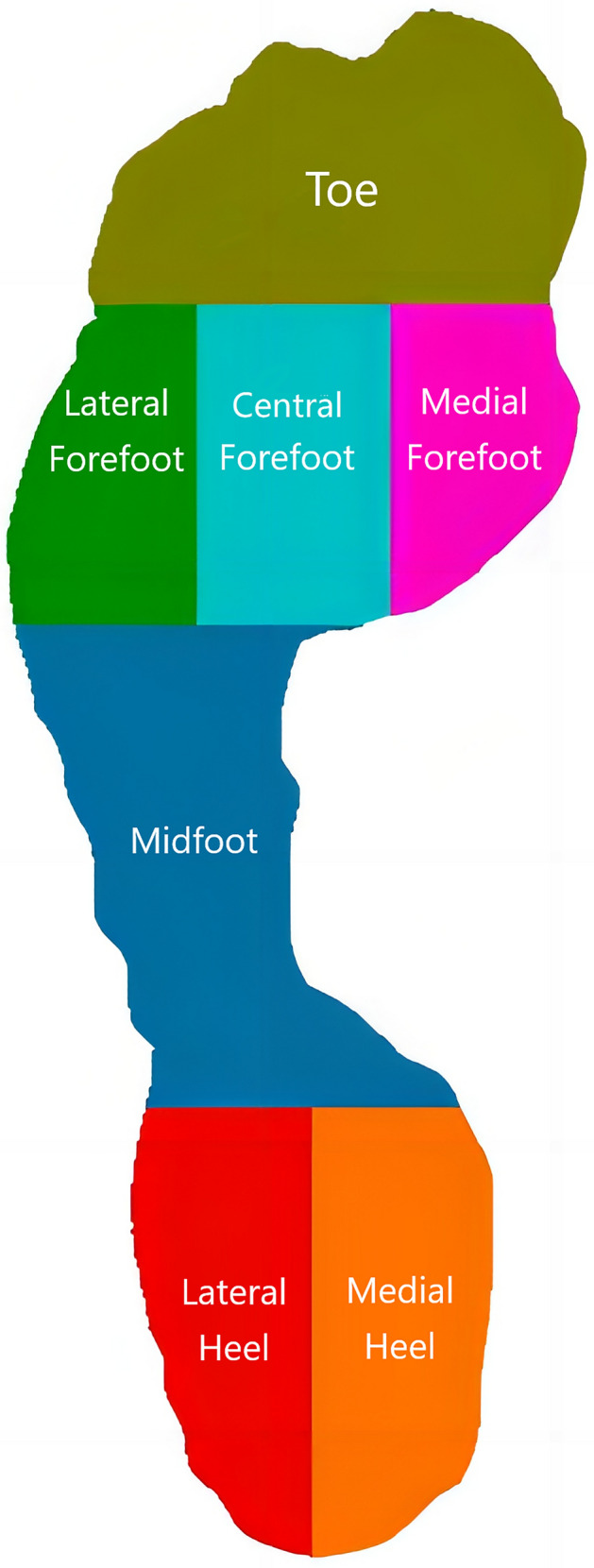
Schematic diagram for the seven subdivided foot zones.

To evaluate plantar loading, the foot's pedobarographic image was compartmentalized into seven designated zones through the following steps (Fig. 1). Initially, the software recognized the foot's profile and bifurcated it into three components: the forefoot, the midfoot, and the hindfoot. Subsequently, the forefoot was divided into two segments horizontally, encompassing the toe area and the metatarsal area. Then, the metatarsal area was distributed into three equal parts vertically, which comprised the medial forefoot (first metatarsal), central forefoot (second and third metatarsals) and lateral forefoot (fourth and fifth metatarsals). Ultimately, the hindfoot was partitioned into two equivalent sections vertically, namely the medial and lateral heels. In order to identify the immediate impact of intoeing for plantar loading during gait, peak pressure and maximum force were chosen, which were normalized by body weight. Additionally, the cumulative effect of plantar loading was assessed by plantar impulse in each foot region. The recorded values were initially averaged for every child and subsequently across 10 children in each group.

### Statistical analysis

Statistical analyses were executed using SPSS 20.0 (IBM Corporation, Armonk, USA). Descriptive statistics, comprising the mean and standard deviation, were computed for each variable, and normality was confirmed via the Shapiro–Wilk test. Pearson's correlation was subsequently utilized to investigate the relationship between FPA and plantar loading variables, including peak pressure, maximum force and plantar impulse for each foot zone. Paired t-tests were conducted to ascertain the impact of FPA on gait symmetry within each group. One-way ANOVA was employed to compare the mild intoeing side among the three groups. Average values were calculated between left and right sides for each subject in group B when performing One-way ANOVA. In instances where the assumption of homogeneity of variances was fulfilled, Bonferroni was applied for multiple comparisons. Otherwise, the Games-Howell post-hoc test was put into use. The results were considered statistically significant when *P* < 0.05.

## Results

### Plantar loading pattern

Significant correlations were observed between FPA and plantar loading variables, including peak pressure, maximum force and plantar impulse, during gait in all three groups, exhibiting particular prominence in group B and group C (Tables 2, 3, 4).

**Table 2:** Correlation between FPA and peak pressure in the seven subdivided foot zones.

Foot zone	Group A						Group B						Group C					
	Normal (N/cm^2^/kg)	r	P	Mild (N/cm^2^/kg)	r	P	Mild-left (N/cm^2^/kg)	r	P	Mild-right (N/cm^2^/kg)	r	P	Mild (N/cm^2^/kg)	r	P	Moderate (N/cm^2^/kg)	r	P
Toe	0.62 ± 0.12	− 0.267	0.456	0.69 ± 0.27	0.303	0.395	0.64 ± 0.11	0.338	0.339	0.61 ± 0.14	0.180	0.618	0.62 ± 0.11	− 0.204	0.572	0.60 ± 0.11	0.374	0.287
Medial forefoot	0.70 ± 0.17	− 0.596	0.069	0.72 ± 0.09	− 0.050	0.891	0.73 ± 0.17	0.645	0.044*	0.73 ± 0.14	0.217	0.547	0.74 ± 0.16	0.342	0.333	0.77 ± 0.21	− 0.174	0.631
Central forefoot	0.58 ± 0.13	− 0.155	0.669	0.57 ± 0.14	0.097	0.790	0.53 ± 0.12	0.700	0.024*	0.57 ± 0.13	0.483	0.157	0.56 ± 0.19	0.634	0.049*	0.57 ± 0.17	− 0.181	0.618
Lateral forefoot	0.57 ± 0.15	− 0.737	0.015*	0.55 ± 0.08	− 0.075	0.836	0.55 ± 0.13	0.112	0.759	0.52 ± 0.14	0.266	0.458	0.56 ± 0.18	0.283	0.428	0.60 ± 0.16	− 0.430	0.215
Midfoot	0.29 ± 0.06	0.108	0.766	0.28 ± 0.04	− 0.108	0.767	0.30 ± 0.08	− 0.180	0.619	0.31 ± 0.11	0.266	0.458	0.33 ± 0.07	− 0.473	0.167	0.36 ± 0.07	0.253	0.480
Medial heel	0.87 ± 0.30	− 0.368	0.295	0.93 ± 0.24	0.233	0.516	0.97 ± 0.27	0.652	0.040*	0.93 ± 0.23	0.080	0.825	0.94 ± 0.41	0.723	0.018*	0.95 ± 0.39	− 0.387	0.270
Lateral heel	0.84 ± 0.29	− 0.319	0.369	0.88 ± 0.20	0.121	0.738	0.92 ± 0.26	0.665	0.036*	0.89 ± 0.22	− 0.031	0.933	0.87 ± 0.34	0.764	0.010*	0.92 ± 0.36	− 0.374	0.287

*Statistically significant at *P* < 0.05.

**Table 3:** Correlation between FPA and maximum force in the seven subdivided foot zones.

Foot zone	Group A						Group B						Group C					
	Normal (N/kg)	r	P	Mild (N/kg)	r	P	Mild-left (N/kg)	r	P	Mild-right (N/kg)	r	P	Mild (N/kg)	r	P	Moderate (N/kg)	r	P
Toe	2.97 ± 0.80	− 0.384	0.274	3.37 ± 1.06	0.411	0.238	3.28 ± 0.84	0.802	0.005*	3.18 ± 0.86	0.051	0.888	3.09 ± 0.29	0.332	0.349	3.10 ± 0.60	− 0.043	0.907
Medial forefoot	1.73 ± 0.75	0.048	0.895	1.91 ± 0.69	0.427	0.218	1.83 ± 0.54	0.851	0.002*	2.01 ± 0.55	0.421	0.225	1.97 ± 0.99	0.680	0.030*	2.09 ± 1.13	− 0.471	0.17
Central forefoot	3.56 ± 1.12	− 0.583	0.077	3.54 ± 0.75	0.317	0.372	3.36 ± 0.80	0.702	0.024*	3.43 ± 0.84	0.253	0.481	3.86 ± 1.53	0.562	0.091	3.96 ± 1.59	− 0.627	0.053
Lateral forefoot	1.94 ± 0.77	− 0.605	0.064	1.80 ± 0.43	0.477	0.164	1.69 ± 0.43	0.516	0.127	1.62 ± 0.47	0.156	0.667	2.02 ± 0.82	0.426	0.219	2.07 ± 1.01	− 0.694	0.026*
Midfoot	2.82 ± 1.01	0.304	0.393	2.59 ± 0.96	0.236	0.512	2.47 ± 0.92	− 0.297	0.404	2.52 ± 0.77	0.234	0.515	3.15 ± 1.01	− 0.061	0.866	2.93 ± 0.90	0.039	0.914
Medial heel	3.86 ± 1.30	− 0.481	0.160	4.17 ± 1.42	0.431	0.213	3.87 ± 0.83	0.705	0.023*	3.91 ± 1.03	0.206	0.567	4.51 ± 1.87	0.753	0.012*	4.20 ± 1.67	− 0.672	0.033*
Lateral heel	3.33 ± 1.17	− 0.227	0.528	3.42 ± 0.99	0.315	0.376	3.54 ± 0.98	0.802	0.005*	3.34 ± 0.76	0.215	0.551	3.75 ± 1.43	0.741	0.014*	3.72 ± 1.58	− 0.718	0.019*

*Statistically significant at *P* < 0.05.

**Table 4:** Correlation between FPA and plantar impulse in the seven subdivided foot zones.

Foot zone	Group A						Group B						Group C					
	Normal (N·s)	r	P	Mild (N·s)	r	P	Mild-left (N·s)	r	P	Mild-right (N·s)	r	P	Mild (N·s)	r	P	Moderate (N·s)	r	P
Toe	11.57 ± 4.83	− 0.461	0.180	13.69 ± 6.58	0.296	0.406	12.74 ± 3.76	0.922	0.001*	13.10 ± 3.76	− 0.077	0.833	11.16 ± 1.38	0.668	0.035*	11.91 ± 2.87	0.034	0.926
Medial forefoot	10.53 ± 4.40	− 0.382	0.276	11.02 ± 4.61	0.438	0.206	9.84 ± 2.96	0.802	0.005*	10.80 ± 3.84	0.329	0.354	11.60 ± 5.72	0.690	0.027*	11.20 ± 5.86	− 0.303	0.394
Central forefoot	19.42 ± 7.92	− 0.664	0.036*	20.35 ± 6.45	0.403	0.249	19.14 ± 5.21	0.704	0.023*	18.80 ± 5.23	0.049	0.892	21.78 ± 7.68	0.654	0.040*	22.82 ± 9.38	− 0.471	0.169
Lateral forefoot	9.13 ± 4.30	− 0.690	0.027*	8.99 ± 2.81	0.489	0.151	8.36 ± 2.87	0.466	0.174	7.57 ± 2.43	− 0.056	0.879	10.47 ± 5.21	0.688	0.028*	9.95 ± 4.10	− 0.601	0.067
Midfoot	13.54 ± 5.76	0.258	0.471	13.27 ± 5.73	0.355	0.313	11.25 ± 4.30	− 0.031	0.932	11.37 ± 3.39	− 0.073	0.840	12.89 ± 5.25	0.417	0.230	13.66 ± 5.57	− 0.579	0.079
Medial heel	18.19 ± 6.75	− 0.274	0.443	18.81 ± 6.14	0.557	0.094	16.34 ± 5.94	0.692	0.027*	16.73 ± 6.42	− 0.027	0.942	18.31 ± 9.36	0.775	0.008*	19.08 ± 10.53	− 0.712	0.021*
Lateral heel	16.40 ± 5.19	− 0.095	0.794	16.30 ± 4.03	0.465	0.176	14.96 ± 4.98	0.792	0.006*	14.30 ± 4.60	− 0.034	0.927	16.72 ± 8.88	0.774	0.009*	17.06 ± 9.27	− 0.732	0.016*

*Statistically significant at *P* < 0.05.

In group A, the FPA exhibited a significant relationship with peak pressure and plantar impulse on lateral forefoot of the normal foot (*r* =  − 0.737, *P* = 0.015 and *r* =  − 0.690, *P* = 0.027) (Tables 2 and 4). Additionally, the impulse in central forefoot zone displayed a negative correlation with FPA on the normal side (*r* =  − 0.664, *P* = 0.036) (Table 4).

In group B, despite the presence of mild intoeing in both limbs, a significant correlation was observed only on the left side. The results indicated a positive correlation between FPA and peak pressure, maximum force, and plantar impulse in four of the seven foot-zones. These zones were medial forefoot ($r = 0.645, P = 0.044,r = 0.851,P = 0.002\,\,{\text{ and }}\,\,r = 0.802,P = 0.005$), central forefoot ($r = 0.700, P = 0.024,r = 0.702,P = 0.024\,\,{\text{ and }}\,\,r = 0.704,P = 0.023$), medial heel ($r = 0.652, P = 0.040,r = 0.705,P = 0.023\,\,{\text{ and }}\,\,r = 0.692,P = 0.027$) and also lateral heel ($r = 0.665, P = 0.036,r = 0.802,P = 0.005\,\,{\text{ and }}\,\,r = 0.792,P = 0.006$) (Tables 2, 3, 4). Moreover, the toe zone also presented a significant correlation with FPA for both maximum force and plantar impulse (*r* = 0.802, *P* = 0.005 and *r* = 0.922, *P* = 0.001) (Tables 3, 4).

For the limb with mild toeing in group C, the impulse in all subdivided foot zone, except the midfoot, showed a positive correlation with FPA. These zones included the toe (*r* = 0.668, *P* = 0.035), medial forefoot (*r* = 0.690, *P* = 0.027), center forefoot (*r* = 0.654, *P* = 0.040), lateral forefoot (*r* = 0.688, *P* = 0.028), medial heel (*r* = 0.775, *P* = 0.008), and lateral heel (*r* = 0.774, *P* = 0.009) (Table 4). Additionally, the heel zone displayed a significant correlation with FPA concerning both peak pressure and maximum force. This was true for both the medial heel (*r* = 0.723, *P* = 0.018 and *r* = 0.753, *P* = 0.012) and lateral heel (*r* = 0.764, *P* = 0.010 and *r* = 0.741, *P* = 0.014) in the limb with mild toeing. There was also a notable correlation between FPA and peak pressure in the central forefoot (*r* = 0.634, *P* = 0.018) as well as maximum force in the medial forefoot (*r* = 0.680, *P* = 0.030) (Tables 2, 3). For the limb with moderate intoeing, FPA showed a significant relationship with maximum force and plantar impulse in both medial heel (*r* =  − 0.672, *P* = 0.033 and *r* =  − 0.712, *P* = 0.021) and lateral heel (*r* =  − 0.718, *P* = 0.019 and *r* =  − 0.732, *P* = 0.016) (Tables 3, 4). Moreover, a negative correlation was observed between FPA and maximum force in the lateral forefoot (*r* =  − 0.694, *P* = 0.026) (Table 3).

No significant differences were identified for peak pressure, maximum force and plantar pressure in any subdivided foot zones among the three groups and also between the two limbs within each group.

Spatiotemporal parameters. There were noticeable differences in several spatiotemporal variables when comparing the severity of intoeing among the three groups. These variables were the loading response phase (*F*(2, 27) = 3.835, *P* = 0.034), single support phase (*F*(2, 27) = 3.438, *P* = 0.047), pre-swing phase (*F*(2, 27) = 3.349, *P* = 0.049) and double support phase (*F*(2, 27) = 3.465, *P* = 0.046) (Table 5). Further post-hoc testing disclosed that group A exhibited longer loading response phase (*P* = 0.011), pre-swing phase (*P* = 0.017), and double support phase (*P* = 0.016), but a shorter single support phase (*P* = 0.021) than group B. When gait symmetry between limbs within each group was compared, no significant differences were found in any spatiotemporal parameters.

**Table 5:** Spatiotemporal variables for the three groups expressed as mean ± SD.

	Group A		Group B		Group C	
	Normal	Mild	Mild-left	Mild-right	Mild	Moderate
Step length	0.76 ± 0.07	0.76 ± 0.10	0.76 ± 0.06	0.81 ± 0.09	0.79 ± 0.06	0.78 ± 0.07
Step time (s)	0.47 ± 0.04	0.47 ± 0.04	0.47 ± 0.05	0.50 ± 0.06	0.45 ± 0.05	0.44 ± 0.04
Loading response phase (%)*	13.10 ± 0.88	12.64 ± 1.19	11.40 ± 1.30	10.56 ± 1.32	11.59 ± 1.63	11.60 ± 1.52
Single support phase (%)*	36.83 ± 1.67	37.53 ± 1.36	37.58 ± 2.00	39.82 ± 1.59	38.03 ± 1.63	38.84 ± 1.79
Pre-swing phase (%)*	12.55 ± 1.20	13.07 ± 0.88	10.72 ± 1.15	11.38 ± 1.22	11.65 ± 1.50	11.60 ± 1.72
Swing phase (%)	37.33 ± 1.24	36.78 ± 1.51	40.17 ± 2.40	39.35 ± 2.28	38.35 ± 1.83	37.89 ± 1.71
Gait width	0.17 ± 0.03		0.16 ± 0.02		0.15 ± 0.02	
Double support phase (%)*	25.67 ± 1.74		22.15 ± 1.94		23.25 ± 2.97	
Gait speed (m/s)	0.93 ± 0.12		0.95 ± 0.12		1.04 ± 0.12	
Gait cadence (steps/min)	128.30 ± 10.50		127.00 ± 11.20		136.20 ± 13.40	

*Statistically significant between group A and B at *P* < 0.05.

## Discussion

The aim of this research was to investigate the potential impact of FPA on gait spatiotemporal and plantar loading patterns in children with unilateral and bilateral intoeing. Our first hypothesis, which proposed a significant correlation between FPA and peak pressure, maximum force and plantar impulse in subdivided foot zones, was largely validated by the bilateral intoeing groups B and C. The second hypothesis, which predicted significant variations in spatiotemporal parameters based on the severity of intoeing, was partially confirmed between groups A and B.

The transfer of body weight follows a particular pattern during the typical gait, being transmitted via the talus to the calcaneus, forwarded along the outer-side foot, traversing the heads of the metatarsal bones towards the inner-side foot, and finally shifting to the great toe^15^. Previous study suggest that the relationship between the FPA and distribution of plantar pressure among normal children is weak^3^. It seems these children are capable of dynamically adjusting their foot loading patterns to offset the influence of FPA. In this current study, except for the central and lateral forefoot zones, no significant correlation was found between FPA and plantar loading within the unilateral intoeing group, which exhibited a pattern similar to that of normal children^3^. The negative relationship between FPA and both peak pressure and plantar impulse in the lateral forefoot of the normal limb implies that as FPA shifts from a normal position to a more pronounced outward angle, both the instantaneous and cumulative loads on the lateral forefoot decrease.This observation is consistent with earlier research, which suggested that an outtoeing gait may serve to augment load bearing in the medial forefoot while simultaneously reducing it on the lateral forefoot^17^.

It comes as somewhat of a surprise that the FPA positively correlated with plantar loading in specific foot zones for only one side (left limb in group B and mild intoeing limb in group C), but not the other limb in these two bilateral intoeing groups. Upon further analysis, it was discovered that FPA primarily influenced the load distribution in the forefoot zone and also the heel zone. With an increase in the severity of intoeing from mild to moderate, the ability of children with bilateral intoeing to make self-adjustments seemed to be somewhat limited. It appears that they attempted to regulate the side that was more severely affected (right limb in group B and moderate intoeing limb in group C), and made concessions for the side that was less affected. Since the right limb is the preferred limb for most children, this may explain why the significant impact of FPA was observed in the left limb rather than the right limb in group B^23^.

Three variables were chosen in the current study, which were peak pressure, maximum force and plantar impulse, to comprehensively evaluate the relationship between FPA and plantar loading distribution. The level of significance for these three correlations did not always align, although the overall trends were consistent. This phenomenon was particularly evident for the side with moderate intoeing in group C, specifically in the lateral forefoot, medial heel, and lateral heel zones (Tables 2, 3). Both peak pressure and maximum force are instantaneous variables that do not consider time. When analyzing the timing of peak pressure and force occurrence, it was noted that these two variables almost coincided during stance phase. Since foot force is calculated by multiplying pressure by the ground contact area, we surmised that the correlation between FPA and maximum force was primarily influenced by the interaction pattern between the foot and the ground, which is affected by internal tibial torsion.

Plantar impulse represents the cumulative effect of force over a given time period while walking. Typically, as gait speed increases, the peak plantar pressure and force in different regions of the foot also increase^24^. However, due to the the shortening of the stance phase with higher gait speed, the plantar impulse may behave differently. As the severity of intoeing intensified, the correlation between FPA and plantar loading pattern became stronger, especially for plantar impulse. This correlation shifted from positive for mild intoeing on the left side in group B and group C to negative for moderate intoeing in group C (Table 4). Therefore, plantar impulse may be a potentially sensitive indicator of abnormal FPA compared to peak pressure and force. Moreover, when moderate intoeing existed, the negative correlation between FPA and both maximum force and impulse in the heel zone indicated that these two subdivided foot regions may consequently sustain secondary damage due to decreased control of deceleration during loading response^25^.

Previous studies have suggested that adults and children were able to shift the plantar load medially and distally during outtoeing gait, and transfer the plantar load laterally and proximally during intoeing gait^3,17^. However, the distribution characteristics of foot loading patterns for intoeing gait appear to lack consistency in the literature, particularly in the forefoot region. Dieter Rosenbaum found that peak pressure and plantar impulse were greater in the lateral forefoot than in the medial forefoot with intoeing gait^17^. In contrast, Li Tingting observed that adolescents with intoeing exhibited significant peak pressures and force concentrated in the central forefoot and heel zones^26^. Our results concur with the latter, suggesting that the maximum force and impulse in the central forefoot are almost double that of the medial and lateral forefoot areas. This could result in excessive strain in this partial area, potentially leading to a collapse of the transverse arch^27^. Moreover, although no significant differences were found in plantar loading pattern among three intoeing groups, an upward trend of up to 20% in both peak pressure and maximum force in the midfoot was observed when comparing group A with group C (Tables 2, 3). This excessive load could impact the height of medial longitudinal arch and ultimately weaken the ability to shift the load from the midfoot to the heel and forefoot^28,29^.

Tibial torsion is not a constant trait from birth, and a certain degree of internal tibial torsion is considered normal during childhood^1^. Prior research concluded that approximately 95 percent of children with intoeing due to internal tibial torsion experienced spontaneous correction before reaching the age of eight^30^. In current study, children with both unilateral and bilateral intoeing exhibited symmetric gaits across their limbs. The presence of symmetry is a crucial factor in forecasting whether spontaneous correction might occur as children grow older^1^. Furthermore, no substantial differences were detected in the spatiotemporal and plantar loading patterns across any subdivided foot zones between the groups, unless the stance phase was segmented into the loading response, single support, and pre-swing phases. These observations suggest that the impact of intoeing on children is limited, even when the severity of intoeing varies between the two limbs. However, as not all children with intoeing will manifest spontaneous correction, the principal challenge lies in identifying those who won't and providing them with the appropriate early intervention to avoid surgical treatment, which is associated with high complication rates^4,31^.

Three limitations existed in our study and should be mentioned. First, the sample size was relatively small for each group, which may hinder the detection of all significant correlations and potential differences among children with intoeing. Second, since this study relied on retrospective data, the age range of children was somewhat limited. Including a larger number of children spanning a broader age range could enhance statistical power and extend the generalizability of the findings to children at different developmental stages. Third, the possible gender effects was not accounted, although previous studies have shown that gender can influence dynamic plantar pressure distribution in early adolescents, young adults, and the elderly^32–34^. However, no significant correlation has been reported between FPA and gender in normal children^3^. Therefore, future research should differentiate between boys and girls to better understand the influence of gender on these findings.

## Conclusion

This study provides insights into how the intensity of intoeing in children can influence gait and plantar loading pattern. The FPA notably correlates with peak pressure, maximum force, and plantar impulse in the medial and central forefoot, as well as the heel zones, in children with bilateral intoeing. Additionally, significant immediate and cumulative plantar loading were also detected in these subdivided foot regions, which potentially increases the risk of injury to related support structures and could contribute to transverse arch deformities. Most of the spatiotemporal parameters did not exhibit significant differences either within or among the groups, except for the subdivided stance phase, suggesting dynamic self-adjustment abilities in children with intoeing to maintain gait symmetry. However, this capacity is constrained when the severity of intoeing intensifies, as demonstrated by the differing FPA correlation results in children with bilateral intoeing. Therefore, assessments of both limbs must be carried out meticulously for children with intoeing, regardless of whether the condition is unilateral or bilateral. It is necessary to conduct a case-by-case analysis to identify children who may not experience spontaneous resolution, allowing for the provision of appropriate early interventions.

## Data Availability

The datasets used and/or analyzed during the current study are available from the corresponding author on reasonable request.
